# Supplementary material for: Early Development of Direct Embryos in the Cultured Anthers of Manihot esculenta Crantz
Source: Plants (Basel). 2020 Oct 6;9(10):1315. doi: 10.3390/plants9101315 (PMC7650799; doi:10.3390/plants9101315)
Supplement: Supplementary file 1 [file plants-09-01315-s001.zip › Table S1.pdf]

S1. SSR markers screened for polymorphisms in donor plant DNA in order to identify the allelic status of anther-derived embryogenic structures of *Manihot esculenta* var. *Kirikawadi.*

	Marker name	Sequence information 5′ -3′	Annealing temperature (°C)
1	SSRY2	CGCCTACCACTGCCATAAAC	58.50
		TGATGAAATTCAAAGCACCA	
2	SSRY4	ATAGAGCAGAAGTGCAGGCG	62.00
		CTAACGCACACGACTACGGA	
3	SSRY8	AGTGGTTTGAGAAGACTGGTGA	57.40
		TTTCCAAAATGGAACTTCAAA	
4	SSRY17	CTTAGAAAAGAAATTGCATGTGAG	58.50
		TGTCTGATCAAGCTGGTGACA	
5	SSRY20	CATTGGACTTCCTACAAATATGAAT	57.80
		TGATGGAAAGTGGTTATGTCCTT	
6	SSRY37	ATGGCAAAAGATCGAGCAAC	60.00
		GGCCAGTAATTCCTCAAGGC	
7	SSRY53	CCATGCAGTAGTGCCATCTTT	59.60
		ATTTTCACCAACCGCAACTC	
8	SSRY64	CGACAAGTCGTATATGTAGTATTCACG	61.35
		GCAGAGGTGGCTAACGAGAC	
9	SSRY69	CGATCTCAGTCGATACCCAAG	60.00
		CACTCCGTTGCAGGCATTA	
10	SSRY73	AAGTTGATGGTTCTGAATCTGGA	60.50
		ACAGTGATTGAGCGAGGCTT	
11	SSRY81	GGCGATTTCATGTCATGCTT	58.50
		TGATTTTCTGCGTGATGAGC	
12	SSRY87	CTCATCTCATGAAGAACTTGTGC	60.00
		AGAGCACGCATTGTGCATTT	
13	SSRY94	AGGATGGACTTGGAGATGGA	60.50
		GGTGGAAGTAAGGCTGTTAGTG	
14	SSRY107	CCATTTTCTCTTGCTTCTGTCA	57.80
		TGGTTTGAAGTCCTATAAAATCCTT	
15	SSRY110	TTGAGTGGTGAATGCGAAAG	60.00
		AGTGCCACCTTGAAAGAGCA	
16	SSRY114	AACAGGAAGGAAAATCAAGCC	57.80
		TCAACTGCAGATTCATTCAAGA	
17	SSRY128	CAGGACATGACGCAATTCTG	58.50
		GCATGTAGAAGTCTTTGCAATTATG	
18	SSRY162	TTTAGTTAGTTGCGCTAGCTTCC	57.80
		AACTCTTAATGGCTAAAATTATTGATG	
