# Supplementary material for: Early Development of Direct Embryos in the Cultured Anthers of Manihot esculenta Crantz
Source: Plants (Basel). 2020 Oct 6;9(10):1315. doi: 10.3390/plants9101315 (PMC7650799; doi:10.3390/plants9101315)
Supplement: Supplementary file 1 [file plants-09-01315-s001.zip › Table S2.pdf]

**S2. Thermal profile used for DNA amplification with cassava SSR primers**

| Step | Temperature ($^{o}C$) | Time (minutes) |
|---|---|---|
| Initial denaturation | 94.00 | 5 |
| | 94.00 | 1 |
| Annealing | Depending on primers | 1 |
| Elongation | 72 | 4 |
| Extension | 72 | 15 |
| Stop | 4 | 2 |
